# Supplementary material for: Energy-dependent protein folding: modeling how a protein folding machine may work
Source: F1000Res. 2021 Jan 5;10:3. [Version 1] doi: 10.12688/f1000research.28175.1 (PMC7898354; doi:10.12688/f1000research.28175.1)
Supplement: Peer review history

# Peer review: Reviewer response for version 1

**DOI:** 10.5256/f1000research.31167.r78384

**Published:** 2021-02-22

**Authors:** José Arcadio Farías Rico

**Affiliations:** 1 Systems and Synthetic Biology Laboratory, Center for Genome Sciences from the National Autonomous University of Mexico, Cuernavaca, Mexico

This short MD publication by Sahakyan *et al.*, proposes an interesting alternative to the classic thermodynamic theory of protein folding in which the polypeptide chain acquires its native conformation spontaneously without any external aid. The authors argue that protein folding must be modelled as an active energy dependent process assisted by a hypothetical folding machine (the ribosome) that applies torsion to the polypeptide chain.

 They present folding trajectories (up to 1500 ns) of five short peptides (15-21 aa) under four different conditions: a) unassisted folding without restrictions and rotation, b) motions restraints to both ends of the peptide, c) a mechanical force is applied to rotate the C-terminus counterclockwise while the N-terminus is restrained and d) a mechanical force is applied to rotate the C-terminus clockwise while the N-terminus is restrained. Most of the peptides adopt an α helical conformation only in the last condition. The authors suggest that the ribosome, and specially the tRNA molecules moving from the A-site to the P-site, apply rotary forces to the nascent chain during cotranslational folding. 

 The idea is interesting and the protein folding community would benefit from the indexing of this study. Currently, it is well established that some proteins fold differently while being synthesized by the ribosome than refolded in diluted buffer conditions. The work acceptably adds to these new ideas, the MD experiments are consistent and its properly presented. This study certainly provides food for thought and it does not intend to present a complete picture of the forces acting on the polypeptide chain during cotranslational folding. 

 The exit tunnel is a complex environment where transient interactions can be stablished between every residue of the growing chain and the macromolecules lining the tunnel. A myriad of factors other than torsion can aid the folding of macromolecules in the tunnel (conformational entropy reduction, transient salt-bridges and Van Der Waals interactions, for instance). Therefore, torsion might play a role but more experiments are needed to fully support this assumption. Also, since the force is applied in the same direction as the alpha helix formation it would definitely accelerate the folding process.

 The weakest point of the publication is the timescale. The rotation (60 degrees/ps) is several orders of magnitud faster than tRNA rotation (1aa/50ms) given a bacterial translation rate of 20aa/s. It would be interesting to see another setup where they rotate the C-terminal residue by 180 degrees only once, and then follow the equilibration of the peptide conformation for some microseconds (helices take microseconds to milliseconds to fold). If the hypothesis is correct one should see the same increased tendency for the peptide to become helical with clockwise rotation compared to counterclockwise as they do under continuous rotation.

 Several further studies are possible, such as testing a bigger number of peptides with different helix propensities to draw statistics on how likely is that torque in the chain produce helix formation versus the effect of the simple reduction on the conformational entropy by confining the peptide in a closed environment (as it is the ribosomal tunnel).

 Also one could try to perform MD with torsion on small protein domains that have already proven to fold very deep of the ribosomal tunnel. There is a very small domain (29 aa) (ref 47) for which its folding has been tested. How difficult is to test the same conditions used for the peptides with this domain? This domain is almost the same size than the peptides and it is a much better representative of a folded domain.

Is the work clearly and accurately presented and does it cite the current literature?

Yes

If applicable, is the statistical analysis and its interpretation appropriate?

Not applicable

Are all the source data underlying the results available to ensure full reproducibility?

Yes

Is the study design appropriate and is the work technically sound?

Yes

Are the conclusions drawn adequately supported by the results?

Partly

Are sufficient details of methods and analysis provided to allow replication by others?

Yes

Reviewer Expertise:

Cotranslational protein folding, biophysics, synthetic biology, protein evolution.

I confirm that I have read this submission and believe that I have an appropriate level of expertise to confirm that it is of an acceptable scientific standard, however I have significant reservations, as outlined above.

# Peer review: Reviewer response for version 1

**DOI:** 10.5256/f1000research.31167.r78915

**Published:** 2021-02-19

**Authors:** Antonio Trovato

**Affiliations:** 1 Department of Physics and Astronomy, University of Padova, Padova, Italy

In this short contribution the authors set out to validate, by means of all-atom molecular dynamics simulations in explicit solvent, the idea that the folding of proteins into their biologically relevant native state depends crucially on the presence of the "in vivo" cellular machinery. They have been arguing through several papers, in recent years, that only a minor subset of short proteins conforms to the widely accepted thermodynamic hypothesis (i.e. the native state is the global free energy minimum for a protein in the test tube). In their view, most proteins are not able to fold "in vitro" and instead evolved to be able to fold while they are synthesized at the ribosome. The energy flow provided by the cellular machinery through mechanical manipulations would than make protein folding an activated process "in vivo".

 Here, in particular, they consider short peptides (around 20 residues) with helical native states. Their molecular dynamics simulations, 1.5 microseconds long, show that folding to the native state rarely occurs in the absence of any restraint. On the other hand, when restraints on the position of the N-terminal residue and a constant clockwise torque on the C-terminal residue are applied, folding to the native helical state readily occurs for all peptides.

 The paper is well written and makes for an interesting read. The authors are very careful in making it clear that any connection between the mechanical restraints used in their simulations and the ones present during co-translational folding is yet to be substantiated. Their findings are in principle interesting since they show how energy pumping through a torque may in fact improve the ability of the folding process to reach a given configuration.

 However, the authors should consider carefully the following remarks, which could undermine, to some extent, their conclusions:

1. I think it is important to check whether the helical native states are stable for the considered all-atom force field in the absence of any restraint (e.g. running a microsecond long simulation with the native state as initial condition); if not, failure to fold into the native state could be more simply ascribed to incorrect force field parametrization. I am assuming that the stability of the native state in solution, at least on the microsecond time scale, should be guaranteed also within the unorthodox view advocated by the authors; a comment by them on this point would be useful.
2. Considering only helical native states may bias the conclusion of this study, due to their definite right-handed chirality. Applying a clockwise torque may be effective in biasing any polymer chain towards adopting a right-handed helical configuration. The authors should then consider, at least for future work, to run simulations for peptides forming beta-hairpins or beta-sheets in their native states.
3. Along the same lines, it had been in fact observed that helical shapes are kinetically favored for growing polymers attached to a moving end ^1^, even in the absence of an explicit torque. My point is again that what found by the authors could be a generic feature shared by any polymer chain, not a property specific to peptide sequences adopting a helical native state.

Is the work clearly and accurately presented and does it cite the current literature?

Yes

If applicable, is the statistical analysis and its interpretation appropriate?

Not applicable

Are all the source data underlying the results available to ensure full reproducibility?

Yes

Is the study design appropriate and is the work technically sound?

Yes

Are the conclusions drawn adequately supported by the results?

Partly

Are sufficient details of methods and analysis provided to allow replication by others?

Yes

Reviewer Expertise:

Computational biophysics; protein folding and aggregation

I confirm that I have read this submission and believe that I have an appropriate level of expertise to confirm that it is of an acceptable scientific standard, however I have significant reservations, as outlined above.

## References

[1] : Form of growing strings. *Phys Rev Lett*.2005;95(9) : 10.1103/PhysRevLett.95.098103 098103 10.1103/PhysRevLett.95.098103 16197255

# Peer review: Reviewer response for version 1

**DOI:** 10.5256/f1000research.31167.r77095

**Published:** 2021-02-02

**Authors:** Stephen D. Fried

**Affiliations:** 1 Department of Chemistry, Johns Hopkins University, Baltimore, MD, USA

In this short article, Sahakyan *et al.* present molecular dynamics (MD) simulations that seek to question the overarching paradigm that protein folding is a predominantly thermodynamically-driven process in which native states are achieved exclusively by identifying states with minimal free energy in the absence of any energy dissipation or physical forces acting on them.

 In contrast, by presenting several µs-length trajectories of several short (20mer) alpha-helix forming peptides, the authors show that the ability for the peptide to rapidly adopt a helical conformation is dependent on the application of an externally applied torque, rotating the C-terminus clockwise. The authors draw the connection that the C-terminus of a nascent protein might also experience rotary forces during co-translational folding induced by the movement of tRNA molecules transiting through the A-site and P-site

 This article presents an interesting and novel idea that the protein folding community should be aware of, as recent years have seen a growing number of contributions highlighting the potential difference between co-translational folding and ‘classical’ folding experiments. I would say that rather than ‘answering’ a problem, it is more an invitation for future work and thought, given the relatively few results that have been reported. That being said, the results presented are intriguing and internally consistent, and deserve the consideration of any worker in the protein folding field interested in understanding how biological folding could differ from the classic scenario of an ergodic search on a constant free energy landscape.

 In evaluating this work, I have a number of conceptual comments, suggestions for further study, and recommendations on reproducibility.

Conceptual comments:

- At several points, the authors mention that the 3’ terminus of the A-site tRNA undergoes a large-scale (ca. 180˚) rotation, and that this forms the basis of their hypothesis that rotary motions could be transduced to a nascent chain during translation. The motion that the authors are referring to – if I’m not mistaken – is the accommodation of the tRNA from the A/T-hybrid state to the A/A-canonical state upon hydrolysis of GTP by EF-Tu (though they do not mention this process by name). My understanding is that the nature of this large rotation *is* understood; it results from the fact that the presence of EF-Tu precludes the ‘top half’ of the tRNA from entering the A-site on the 50S subunit whilst the anticodon engages with the decoding center; release of EF-Tu then allows the 3’-end of the A-site tRNA to swing into the PTC. That being said, this large movement would not be experienced by the nascent chain *per se*, because it is occurring on the A-site tRNA (not the P-site tRNA carrying the nascent chain). Hence, the authors may need to clarify what rotational conformational changes are experienced by the P-site tRNA, and provide structural data that directly support this.
- Is the rate of the rotary movement relevant to the timescale of translation? If not, this should be commented on directly, or at least probed by varying its rotational frequency.

Further study:

- Seeing as the larger rotational movement is associated with the A-site tRNA (before its aminoacylated acid is added to the peptidyl chain), the authors may want to use simulation to directly test their hypothesis that “ *this motion might lead to the rotation of the C-terminus of the nascent peptide*.” I appreciate the challenge involved in such, as it would surely require performing relatively long simulations on the ribosome. Though this would represent a truly important contribution and should be considered. If simulations of this size/timescale are too infeasible, potentially a careful analysis of the many ribosome structures with P-site tRNA-nascent chains bound could also serve as a reasonable way to interrogate this question.
- The authors seem to assume that all the peptides they studied *could* become alpha helical under the present forcefield, and that this is accelerated by the presence of the external torque. I suppose the first part of this should probably be shown explicitly, perhaps by a free-energy perturbation or umbrella sampling approach, just to show that the minimum free-energy states are in fact what we would think they are.
- What is the mechanism whereby the rotary force induces folding? Careful analysis of the trajectories could probably support or refute the hypothesis that it helps ‘seed’ the formation of the alpha helix, which then ‘zippers’ up. (Actually the zippering model for alpha-helix formation is probably very relevant to the authors’ discussion, and they might consider seeing if their simulations reproduce the parameters from classical statistical mechanical treatments of the helix-coil problem).

Reproducibility:

- Seeing as performing MD simulations in the presence of external torques is fairly non-standard, the authors may want to provide the actual GROMACS input files for any computational biochemist interested in performing similar simulations.

Is the work clearly and accurately presented and does it cite the current literature?

Yes

If applicable, is the statistical analysis and its interpretation appropriate?

Yes

Are all the source data underlying the results available to ensure full reproducibility?

Yes

Is the study design appropriate and is the work technically sound?

Yes

Are the conclusions drawn adequately supported by the results?

Partly

Are sufficient details of methods and analysis provided to allow replication by others?

Partly

Reviewer Expertise:

Protein folding biophysics; proteomics and mass spectrometry.

I confirm that I have read this submission and believe that I have an appropriate level of expertise to confirm that it is of an acceptable scientific standard, however I have significant reservations, as outlined above.

# Peer review: Reviewer response for version 1

**DOI:** 10.5256/f1000research.31167.r76648

**Published:** 2021-01-25

**Authors:** Lisa J. Lapidus

**Affiliations:** 1 Department of Physics and Astronomy, Michigan State University, East Lansing, MI, 48824, USA

This paper presents an intriguing hypothesis, that torque applied by the ribosome at the A-site, actively assists folding. To demonstrate this, the folding of 5 small proteins were simulated from an extended state. When clockwise torque is applied to the C-terminus while constraining the N-terminus (so that the torque is maintained within the backbone), the proteins typically folded within 1.5 microseconds and did not fold within that window without torque, without restraint, and if the torque was applied counter-clockwise. 

 The results are quite convincing but a few details are missing. Below are some questions whose answers would add to the understanding of the reader:

 1. What happens to the backbone after the torque is applied? It seems like the N-terminus is constrained for the entire length of the simulation but this should be made more clear. 

 2. How does the collapse of the backbone proceed with the constraints compared to without?

 3. Do the helical segments form simultaneously or do helices propagate down the length of the chain? Another figure showing native structures of the sequences color coded by when native structure is formed might by useful (or something equivalent).

 This paper has an interesting premise with a lot of caveats given the simplified results supporting the conclusion. However, the authors do a good job of addressing some of them. I am not completely convinced that crowded cell conditions could provide the N-terminal constraint required to acquire structure rapidly, but this is a testable result for current co-translational folding simulations. The authors discuss that the torque applied by the ribosome tunnel is a open question and may not be mimicked by the simple torque in the simulations. Another question that could be answered by more realistic simulations is what is the effect of torque at the A-site on the nascent chain outside the ribosome tunnel. While helices can form in the tunnel, there is not a lot of evidence that helices emerge from the tunnel for most sequences. So is the net torque on the last part of the chain before leaving the tunnel what really matters?

Is the work clearly and accurately presented and does it cite the current literature?

Yes

If applicable, is the statistical analysis and its interpretation appropriate?

Not applicable

Are all the source data underlying the results available to ensure full reproducibility?

Yes

Is the study design appropriate and is the work technically sound?

Yes

Are the conclusions drawn adequately supported by the results?

Partly

Are sufficient details of methods and analysis provided to allow replication by others?

Yes

Reviewer Expertise:

I study protein folding using ultra-fast experimental methods. I have pioneered the method of measuring intramolecular diffusion of unfolded proteins leading to the estimates of protein folding speed limits.

I confirm that I have read this submission and believe that I have an appropriate level of expertise to confirm that it is of an acceptable scientific standard.
